# Sample size considerations using mathematical models: an example with *Chlamydia trachomatis* infection and its sequelae pelvic inflammatory disease

**DOI:** 10.1186/s12879-015-0953-5

**Published:** 2015-06-19

**Authors:** Sereina A Herzog, Nicola Low, Andrea Berghold

**Affiliations:** Institute for Medical Informatics, Statistics and Documentation, Medical University of Graz, Graz, Austria; Institute of Social and Preventive Medicine, University of Bern, Bern, Switzerland

**Keywords:** Sample size calculation, Mathematical model, Compartmental model, Randomised controlled trials, Chlamydia infection, Pelvic inflammatory disease

## Abstract

**Background:**

The success of an intervention to prevent the complications of an infection is influenced by the natural history of the infection. Assumptions about the temporal relationship between infection and the development of sequelae can affect the predicted effect size of an intervention and the sample size calculation. This study investigates how a mathematical model can be used to inform sample size calculations for a randomised controlled trial (RCT) using the example of *Chlamydia trachomatis* infection and pelvic inflammatory disease (PID).

**Methods:**

We used a compartmental model to imitate the structure of a published RCT. We considered three different processes for the timing of PID development, in relation to the initial *C. trachomatis* infection: immediate, constant throughout, or at the end of the infectious period. For each process we assumed that, of all women infected, the same fraction would develop PID in the absence of an intervention. We examined two sets of assumptions used to calculate the sample size in a published RCT that investigated the effect of chlamydia screening on PID incidence. We also investigated the influence of the natural history parameters of chlamydia on the required sample size.

**Results:**

The assumed event rates and effect sizes used for the sample size calculation implicitly determined the temporal relationship between chlamydia infection and PID in the model. Even small changes in the assumed PID incidence and relative risk (RR) led to considerable differences in the hypothesised mechanism of PID development. The RR and the sample size needed per group also depend on the natural history parameters of chlamydia.

**Conclusions:**

Mathematical modelling helps to understand the temporal relationship between an infection and its sequelae and can show how uncertainties about natural history parameters affect sample size calculations when planning a RCT.

**Electronic supplementary material:**

The online version of this article (doi:10.1186/s12879-015-0953-5) contains supplementary material, which is available to authorized users.

## Background

In the field of infectious diseases, planning the required sample size for an intervention trial raises special issues. The success of an intervention to prevent the disease complications by reducing exposure to infection is influenced by the natural history of the infection. Different assumptions about the temporal relationship between the infection and the development of sequelae can affect the expected effect size of an intervention. To calculate the required sample size for a trial investigating the reduction in complications of an infection, we need to make assumptions about the effect size of the intervention (e.g. the reduction in relative risk) and the incidence of the infection sequelae.

Mathematical models are often used to study the dynamics of infectious disease transmission. Researchers have also suggested that mathematical models could help to improve the design of randomised controlled trials (RCTs) of complex interventions to prevent infectious disease transmission [1–4]. In a recent example, a deterministic compartmental model was developed to inform the design and monitoring of the HIV Prevention Trials Network study PopART (HPTN 071). PopART is a three-arm cluster-randomized trial to investigate the effectiveness of antiretroviral therapy and other interventions to reduce HIV transmission at the population level [5, 6]. Mathematical models are particularly useful for investigating processes and mechanisms that are difficult to observe in practice [3], e.g. the development of complications of infections that might be diagnosed sometime after exposure to the infection [7]. The effect size of an intervention can be derived within the mathematical modelling framework. The results obtained for the effect size can then be used to determine the required sample size.

*Chlamydia trachomatis* (chlamydia) infection in the lower genital tract can ascend to cause pelvic inflammatory disease (PID) in women which, in turn can cause ectopic pregnancy and tubal factor infertility. Chlamydia is the most common bacterial sexually transmitted infection in many developed countries [8, 9] and is mostly asymptomatic in women, but treatable with antibiotics if diagnosed [10]. There is still considerable uncertainty about how long after initial infection *C. trachomatis* ascends to the upper genital tract, resulting in PID, and the fraction of infected women who will develop PID [11–13].

There is great interest in interventions that could reduce the risk of chlamydia-associated PID because this might prevent future tubal factor infertility. The Prevention Of Pelvic Infection (POPI) RCT investigated the effect of offering young women in London a screening test for chlamydia on the incidence of PID one year later [14, 15]. Women were randomly allocated to an intervention group, which received immediate testing and treatment for women with positive chlamydia test results. The control group reflected routine care, but swabs were collected at baseline and stored. Testing and treatment were then deferred for one year. The incidence of PID in the trial was lower in the intervention than the control arm but the confidence intervals included the null effect and the investigators argued that the trial was underpowered [14, 15]. We used published data and assumptions from the POPI trial to: investigate how different assumptions about the temporal relationship between chlamydia infection and the development of PID can influence the sample size calculation; and to investigate how a mathematical model can be used to inform sample size calculations for an RCT.

## Methods

### Model

**Fig. 1:**
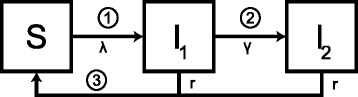
Schematic overview of the model framework. The Susceptible-Infected-Susceptible (SIS) compartmental model allows investigating three hypothetical temporal relation assumptions between chlamydia infection and PID. A susceptible woman (S) can become infected (I) at a constant rate *λ* and can clear the infection naturally at rate *r*. Numbers indicate when during the chlamydia infection progression to PID could happen: 1) immediate progression, 2) constant progression, and 3) progression at the end. For the constant progression, an infected woman can progress at rate *γ* from being infected without PID (I_1_) to being infected with PID (I_2_). We set *γ* = 0 and I = I_1_ + I_2_ for immediate progression and progression at the end. PID, pelvic inflammatory disease.

We used a Susceptible-Infected-Susceptible (SIS) compartmental model (Fig. 1) to imitate the structure of the POPI RCT. We ran the model separately for intervention and control groups, using different starting conditions because chlamydia status at baseline differed in the intervention and in the control group (Additional file 1, section 1) [16]. Women in the intervention group are all initially susceptible (S) because they have been tested for chlamydia and received (presumed) successful treatment if indicated. In the control group, a percentage of women is infected (I), reflecting the chlamydia prevalence in the study population. We used a constant force of infection, assuming that a single screening test in women participating in the RCT did not change the population prevalence of chlamydia, i.e. susceptible women can become infected at a constant rate *λ*. Infected women can clear the infection naturally at rate *r*. We investigated differences in the temporal relationship between chlamydia infection and PID, as described previously, by separating the infectious period into two stages [16]. The first stage represents infected women without PID (I_1_) who can progress at rate *γ* to the second stage where the infected women have developed the complication PID (I_2_). This results in the following system of ordinary differential equations:

$$ \frac{dS(t)}{dt}=-\lambda S(t)+r\left({I}_1(t)+{I}_2(t)\right) $$

$$ \frac{d{I}_1(t)}{dt}=\lambda S(t)-\left(r+\gamma \right){I}_1(t) $$

$$ \frac{d{I}_2(t)}{dt}=\gamma {I}_1(t)-r{I}_2(t) $$

The force of infection *λ* is calculated so that the steady state prevalence in the model is equal to the prevalence *p* of the study population. The duration of infection is assumed to be exponentially distributed with a mean duration of 1/*r*, i.e. some women will clear the infection rapidly whereas others remain infected for longer time periods [17, 18].

### PID incidence and intervention effect for three types of progression

We examined three hypothetical mechanisms for the timing of progression from chlamydia infection to PID [16]: first, PID could develop soon after *C. trachomatis* infects the lower genital tract (immediate progression); second, PID could develop at any time throughout the course of lower genital tract infection (constant progression); or third, PID could develop just before natural clearance from the lower genital tract (progression at the end). For each type of progression it is assumed that, of all women infected, a certain fraction *f* will develop PID in the absence of an intervention (Fig. 1). If there is immediate progression the PID incidence equals *fλ*S, we set *γ* = 0 and I = I_1_ + I_2_. If PID develops at a constant rate the PID incidence equals *γ*I_1_; to achieve the same cumulative PID incidence as the other two types of progression we set $\gamma =\frac{fr}{1-f}$. Note, the mean duration of infection is still 1/r (see Additional file 1, section 1). If PID develops at the end of a chlamydia infection PID incidence equals *fr*I setting *γ* = 0 and I = I_1_ + I_2_.

In the absence of an intervention, the PID incidence is the same for all three types of progression, i.e. the cumulative PID incidence rates in the control group at follow-up time *t* are the same. The cumulative PID incidence in the intervention group at follow-up time *t*, derived for each type of progression by the model, is used to calculate the corresponding relative risk (RR) of PID (RR = risk of PID in intervention group/risk of PID in control group). The RR is independent of the fraction *f* for the immediate progression and for the progression at the end in contrast to the RR for the constant progression (Additional file 1, section 2).

### Sample size calculations used in the POPI trial

The sample size calculation for the POPI trial was based on a comparison of two proportions. Standard formulae used to calculate the sample size need assumptions about the PID incidence in both groups after the follow-up period, or about PID incidence in the control group and the RR (Additional file 1, section 3) [19]. The POPI trial investigators published two sample size calculations (Table 1). Before the start of the trial, they assumed a 2 % PID incidence after one year in the control group and RR = 0.48 resulting in a total sample size needed of 4122 (with 80 % power and a 5 % significance-level), if there was no loss to follow up, based on a published RCT. In that RCT, 7 % of women in the intervention group had a positive chlamydia test result at baseline and the incidence rates of PID one year later were 8 per 10,000 woman months in the intervention and 18 per 10,000 woman months in the control group (RR 0.44, 95 % CI 0.20-0.90) [20]. During the POPI trial, the investigators revised their sample size calculation, owing to slow enrolment. The revised calculation cited a cohort study suggesting a higher PID incidence (9.5 % to 12.0 % over four years in three different groups of women) [21]. In the revised calculation, they assumed a PID incidence of 3 % and calculated the sample size needed to detect a RR of 0.44; a sample size of 2274 women would be required to detect this effect size with 80 % power and a 5 % significance level [14, 15].

**Table 1:** Parameter values describing sample size calculation, the natural history of chlamydia infection and PID development

Parameters		Re-examine POPI trial		Generic sample size calculation		Source
		Baseline values	Range	Baseline values	Range	
*Sample size calculation*						
*inc*	PID incidence (per year)	1) 2 % & 2) 3 %^a^		calculated^†^		POPI trial [14]
*RR*	Relative risk	1) 0.48 & 2) 0.44^a^		calculated^a^		POPI trial [14]
*t*	Follow-up time (in days)	365		365	90–540^‡^	POPI trial [14]
*α*	Significance level	5 %		5 %		POPI trial [14]
1-*β*	Power	80 %	10–90 %^†^	80 %		POPI trial [14]
*Infection parameter*						
*λ*	Force of infection (per day)	calculated^§^		calculated^§^		
1/*r*	Duration of infection (in days)	365	365 ± 75	365	365 ± 75	Model [17]^¶^
*p*	Prevalence of infection	7 %	3–10 %^‡^	7 %	3–10 %^‡^	POPI trial [14]
*Progression to PID*						
*f*	Fraction of women with chlamydia who progress to PID in absence of testing	calculated^†^		10 %	7–13 %	Model [16]^¶^
1/*γ*	Infection progression (in days)	calculated^#^		calculated^#^		

^a^For the three types of progression, PID incidence is used for the control group and RR is calculated per type

^†^The fraction *f* and the PID incidence *inc* in the control group satisfy the equation: *fprt* = *inc*

^‡^Range determined by agreement among authors

^§^In the absence of the trial, to observe chlamydia prevalence *p* at steady state: $\lambda =\frac{pr}{1-p}$

^¶^Results of a mathematical model which used published trial data

^#^To achieve the same cumulative PID incidence in all three processes in absence of the trial: $\gamma =\frac{fr}{1-f}$

PID, pelvic inflammatory disease; RR, relative risk

In this study, we first re-examined the data used for the two sample size calculations in the POPI trial:

- Scenario 1, 2 % PID incidence and RR = 0.48;
- Scenario 2, 3 % PID incidence and RR = 0.44.

We determined the fraction *f* such that the PID incidence in the control group after the follow-up period equals the PID incidence assumption using baseline values for all other parameters (Table 1). For each type of progression from chlamydia to PID, we used the model to derive the incidence of PID in intervention and control groups, and the corresponding RR. We used the standard formula for calculating the sample size and compared the results with the original calculation, varying the power of the trial from 10–90 %. A sensitivity analysis for scenario 1 was done by varying duration of infection and prevalence, keeping baseline values for all other parameters.

### Generic sample size calculation using the mathematical model

In a second step, we investigated how the mathematical model can inform sample size calculations in general. We used the model and the three different types of progression to examine sample size requirements using different values for the natural history parameters of chlamydia infection instead of assuming a specific PID incidence in the control group.

First, we used baseline values for all parameters (Table 1) to derive the PID incidence in the control group, the RR for each type of progression and the sample size needed per group. We then changed the duration of infection and fraction developing PID using baseline values for all other parameters (Table 1). The analysis was repeated for different values of chlamydia prevalence. We also investigated the change of PID incidence in the control group and the RRs while varying the follow-up time from three to 18 months using baseline values for all other parameters (Table 1).

For all analyses, the sample size needed per group was calculated using the chi-square test for the comparison of two proportions [19]. Analytical results were derived in Mathematica 10 and numerical solutions were obtained in R (version 3.1.1) [22, 23]. Code files can be obtained from the authors on request. This study used published data only so it did not require approval by an ethical committee.

## Results

### Sample size calculations used in the POPI trial

**Fig. 2:**
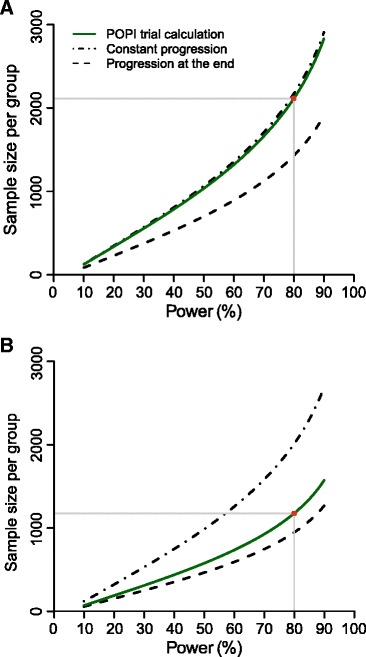
Estimated sample sizes under the two assumptions in the Prevention Of Pelvic (POPI) trial. Plotted curves represent the estimated sample size needed per group while varying power of the study; for the original POPI trial (green lines) and for two types of progression; the one where PID develops at a constant rate throughout infection (dashed-dotted lines), and the one where PID develops at the end of infection (dashed lines). The third type of progression where PID develops immediately after infection is not shown. Panels **a** and **b** separate the two different assumptions about the projected follow-up incidence of PID in the original POPI trial: scenario 1 with 2 % per year (Panel **a**), and scenario 2 with 3 % per year (Panel **b**). The red circle represents the sample size with 80 % power in the original POPI trial. PID, pelvic inflammatory disease; POPI trial, Prevention Of Pelvic Infection trial

Different sets of published assumptions about PID incidence rates and the size of intervention effect lead to different conclusions about the temporal relationship between chlamydia infection and PID (Fig. 2). In scenario 1, the relationship between the power of a trial and the sample size required per group is compatible with the hypothesis that PID can develop throughout the course of infection (Fig. 2a). Assuming a constant progression rate from chlamydia to PID results in a RR of 0.49, which is close to the original RR assumption in the POPI trial (RR = 0.48). If chlamydia progresses to PID only at the end of the infectious period, the model predicts a RR of 0.39. In this scenario, 28.6 % of infected women have to develop PID to achieve the 2 % PID incidence after one year of follow-up.

In scenario 2, the assumptions used for the second sample size calculation (RR = 0.44, 3 % PID incidence) are more compatible with the hypothesis that PID develops at the end of a chlamydia infection (Fig. 2b). This model results in a RR of 0.39, as before. In a model that assumes a constant progression rate from chlamydia to PID, the predicted RR = 0.56. In this scenario, 42.9 % of chlamydia-infected women have to develop PID to achieve an incidence rate of 3 % after one year of follow-up. The RR and the sample sizes needed per group are always higher for constant progression to PID than for progression at the end of infection (Fig. 2). If chlamydia infection progresses to PID only at the end of infection, infection can resolve naturally before they are at risk of PID, whereas with constant progression the risk of developing PID remains throughout the infectious period.

Under the hypothesis that *C. trachomatis* results in PID almost immediately after infection, the sample size needed per group is >21,000 for the examined range of power (data not shown) and the predicted PID incidence in the intervention group is higher than in the control group (RR = 1.05 in both scenarios). In this situation, testing and treatment are too late to prevent any PID cases. On the contrary, women who are successfully treated in the intervention group are at risk to become newly infected and to develop PID again.

In the sensitivity analysis for scenario 1, the fraction of women who develop PID in order to achieve the 2 % PID incidence ranged from 15.9 to 80.3 % at different levels of chlamydia prevalence and duration of infection (Additional file 1: Figure S2). For constant progression and progression at the end of infection, changing duration of infection influences the RR and the sample size needed per group more than changing prevalence (see Additional file 1: Table S1 and Figure S3). The sample size calculated with constant progression is closer to the POPI trial sample size calculation than with progression at the end of infection in 78.5 % of the investigated combinations (see Additional file 1: Figure S4). The hypothesis of immediate progression was not investigated because the estimated RR was >1 in the main analysis.

### Generic sample size calculation using the mathematical model

Assuming a chlamydia prevalence of 7 %, a mean infection duration of one year, and that 10 % of infected women will develop PID, we expect a PID incidence of 0.007 after one year of follow-up. Table 2 shows the resulting RR and the sample size needed per group for each type of progression from chlamydia infection to PID. For example, if PID occurs at a constant progression rate, PID incidence is 0.007 per year in the control group and 0.0029 per year in the intervention group. A chi-squared test with a 5 % two-sided significance level will have 80 % power to detect this RR of 0.42 with a sample size of 4654 women in each group. With this sample size we would expect 33 PID cases in the control group and 14 PID cases in the intervention group after one year of follow-up.

**Table 2:** Example for sample size calculation

Type of progression	PID incidence (per year) in control group^a^	Relative risk^a^	Sample size needed per group
Immediate progression	0.007	1.05	1,071,082
Constant progression	0.007	0.42	4,654
Progression at the end	0.007	0.39	4,123

^a^Baseline values of Table 1 ‘General sample size calculation’ have been used for all types of progression

PID, pelvic inflammatory disease

**Fig. 3:**
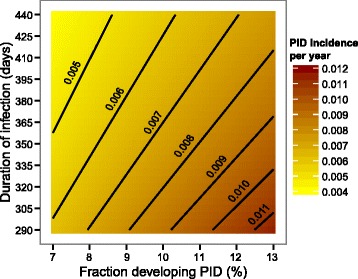
PID incidences per year varying duration of infection and fraction developing PID. The contour plot presents the PID incidences per year while varying infection duration and fraction of women developing PID. The contour lines show for which combinations the PID incidence remains the same. For all other model parameters the baseline values were used (Table 1, Generic sample size calculation). PID, pelvic inflammatory disease.

**Fig. 4:**
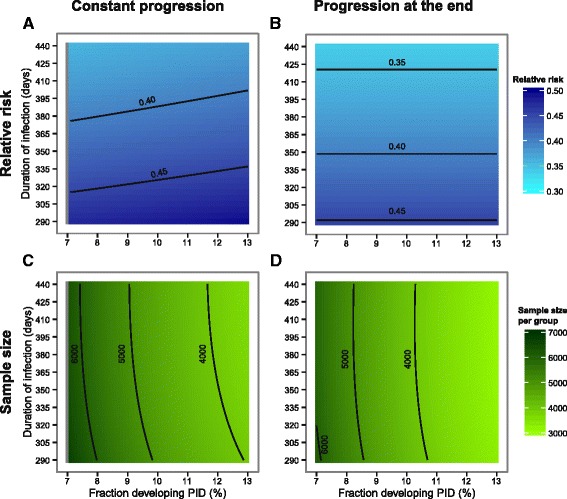
Relative risk and sample size per group varying duration of infection and fraction developing PID. The contour plots present the estimated relative risk (RR) and the corresponding sample size needed per group for the constant progression (Panel **a** and **c**) and for the progression at the end (Panel **b** and **d**) while varying infection duration and fraction of women developing PID. The contour lines show for which combinations the RR and the sample size remain the same. For all other model parameters the baseline values were used (Table 1, Generic sample size calculation). Note that the RR and sample size needed per group cannot be estimated if the fraction of women who develop PID equals the prevalence and progression to PID is constant because this results in a division by zero in the RR formula (indicated by the grey area in **(a)** and (**c**))

Varying the duration of infection from 290 to 440 days and the fraction of women who develop PID from seven to 13 % results in a median PID incidence of 0.007 per year (range 0.0041–0.0115, Fig. 3). The incidence of PID decreases with increasing duration of infection because fewer women in the control group become newly infected during the follow-up period. PID incidence increases with an increasing fraction of women who develop PID. Figure 4 shows the resulting RR and the sample size needed per group for constant progression and progression at the end. Although the RR decreases with increasing duration of infection (Fig. 4a and 4b), the sample size needed per group is almost unaffected by the change in duration of infection (Fig. 4c and 4d) or by changing chlamydia prevalence (not shown).

For constant progression from chlamydia infection to PID, the median RR is 0.42 (range 0.36–0.49, Fig. 4a) with a median sample size needed per group of 4,667 (range 3,644–6,657, Fig. 4c). For progression at the end, the median RR is 0.39 (range 0.34–0.45, Fig. 4b) and the corresponding sample size needed per group has a median of 4,149 (range 3,154–6,125, Fig. 4d). The RR resulting from immediate progression is >1 and the sample size needed per group is >800,000 (see Additional file 1: Figure S5).

The sample size needed per group depends on PID incidence in the control group and the RR. Hence, the observed pattern of sample size needed for different values of the infectious duration and fraction developing PID is a combination of the estimated patterns for PID incidence and RR. We therefore investigated the relation between sample size needed per group, RR, and PID incidence while varying the fraction developing PID. For the hypotheses of immediate progression to PID and progression to PID at the end of chlamydia infection, the RR is independent of the fraction developing PID, i.e. increasing the fraction developing PID increases PID incidence and decreases the sample size needed per group if everything else is kept constant. For constant progression to PID the relationship between sample size needed per group and the fraction developing PID is more complicated because the RR also depends on the fraction developing PID. In this situation, PID incidence and RR need to be investigated together to predict the sample size needed per group while varying the fraction developing PID (see Addition file 1, section 4).

**Fig. 5:**
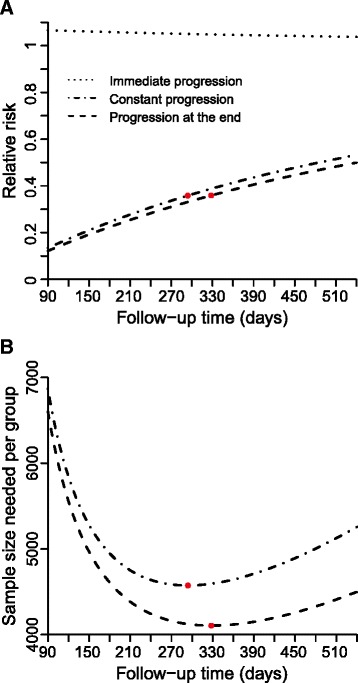
Relative risk and sample size per group varying follow-up time. Plotted curves represent the relative risk (Panel **a**) and the corresponding sample size needed per group (Panel **b**) while varying the follow-up time using for all other model parameters the baseline values (Table 1, Generic sample size calculation): immediate progression (dotted line); constant progression (dashed-dotted line); and progression at the end (dashed line). The red circles indicate the follow-up time which minimises the sample size needed per group for the constant progression and progression at the end

The last analysis in our study showed that there are optimal follow-up times which minimise the sample size needed per group if all infectious parameters and the fraction developing PID are fixed (Fig. 5). For constant progression to PID, a follow-up time of 294 days minimises the sample size needed to 4574 per group with a RR of 0.358 and a PID incidence of 0.0056 per year. For progression to PID at the end, a follow-up time of 328 days minimises the sample size needed to 4106 per group with a RR of 0.359 and a PID incidence of 0.0063 per year. The immediate progression hypothesis was not further investigated because RR > 1.

## Discussion and conclusion

This study showed how a mathematical model can be used to inform sample size considerations for RCTs. We used the example of a screening intervention to prevent chlamydia infection and PID as a complication by re-examining published sample size calculations from the POPI trial. Different sets of assumptions about PID incidence and the RR values used for the sample size calculation in the POPI trial required different hypotheses about the temporal relationship between chlamydia and PID. The sample size calculation in the POPI trial using 2 % PID incidence per year and RR = 0.48 was compatible with an assumed constant progression rate from chlamydia infection to PID, whereas with 3 % PID incidence and RR = 0.44 progression from chlamydia to PID would have to occur at the end of the infectious period. In addition, using the outputs of our model in a generic sample size calculation we found large differences in the required sample size between the POPI trial calculation and our model prediction. For example, with a constant progression rate, the median total sample size required was 9,334 and ranged from 7,288 to 13,314, depending on the values of the infection parameters. This is more than double the amount considered by the POPI trial investigators in their first calculation. Not to forget, the RR and the corresponding sample size needed per group depend also on the other natural history parameters of the infection, however, not all infection parameters have the same impact.

The strength of this study was the exploration of three different temporal relationships between chlamydia infection and PID within the same modelling framework. The model does, however, make several simplifying assumptions. First, the three types of progression are hypothetical because the immunological and pathogenetic mechanisms of PID development are still uncertain [11–13]. It is not biologically plausible that PID develops either immediately after *C. trachomatis* infection or just before natural clearance; they represent the extreme consequences of early and late progression [16]. Nevertheless, we found that the type of progression was an important factor in the sample size consideration. Second, the model does not include treatment failure for the intervention group, which would reduce the effect size and result in an increase in sample size. Third, we assumed no change in chlamydia test behaviour during the trial, which could either increase or decrease the effect size. Fourth, we considered a closed population, i.e. no loss-to follow-up, so this would have to be factored into a sample size calculation. Finally, we focused on a SIS model but including an immunity stage did not alter the results in our study about the sample size considerations in the POPI trial (Additional file 1, section 7).

To our knowledge this is the first study to investigate how a mathematical model can be used for sample size calculations in trials of chlamydia infection and PID prevention. Few mathematical modelling studies explicitly state assumptions about the timing of progression from chlamydia to PID, despite their importance in understanding chlamydia infection and disease [7]. Smith and colleagues examined the impact of different intervals between chlamydia infection and PID development on the cost-effectiveness of chlamydia screening [24]. Tuite and colleagues incorporated an assumption that PID develops at the midpoint of *C. trachomatis* infectious period to estimate the burden of chlamydial infection [25]. Gray and colleagues assumed a uniform rate of progression from chlamydia infection to PID in their investigation of the effects of a chlamydia vaccine [26]. The advantage of our approach is that we have compared the implications of different assumptions for RCT design and planning.

The POPI trial data and our modelling study together allow an interpretation of the postulated temporal relationship between chlamydial infection and PID in the context of published RCT evidence. The first set of assumptions used to calculate the required sample size (chlamydia prevalence 7 %, PID incidence 2 %, RR 0.48) was compatible with the hypothesis that PID can occur at any time during the infectious period of *C. trachomatis*. This mechanism is supported by the effects of similar screening and treatment interventions in other RCTs [15, 20, 27]. The observed chlamydia prevalence and PID incidence in the POPI trial were close the actual assumptions but the effect size was smaller, so the trial was underpowered. The second set of assumptions was quite similar (chlamydia prevalence 7 %, PID incidence 3 %, RR 0.44) but the mathematical model showed that these conditions could only be satisfied if *C. trachomatis* progresses to PID at the very end of the infectious period. This hypothesis lacks biological plausibility, given that chlamydial infectious load in the lower genital tract should be lower at the end than the beginning of infection [26]. The results of published RCTs also argue against immediate progression to PID after *C. trachomatis* infection [15, 20, 27]. Our model allowed us to examine the probability of chlamydia infection progressing to PID. In our model, both sets of sample size assumptions would require a high fraction of chlamydia infection progressing to PID (28.6 % with yearly PID incidence of 2 % and 42.9 % PID incidence 3 %). Estimates of this size have been used in several cost-effectiveness studies [7]. However, two recent modelling studies analysing the results of the POPI trial estimated that 10 % (95 % CI 7–13 %) respectively 12 % (95 % CrI 2–24) of women develop PID [16, 28].

Our approach can be applied to other infections and diseases. The mathematical model introduced in this study could be adapted to other sexually transmitted infections such as *Mycoplasma genitalium*, for which there is great uncertainty about the natural history, but for which screening interventions have been advocated [29]. The model can also be extended to incorporate different types of infection (e.g. those for which immunity after infection is more important) and other assumptions about the temporal relationship between the infection and the complication. This study has implications for the future planning of RCTs. The relationship between an infection and its disease complications need to be understood before planning intervention trials. The findings of this study suggest that mathematical modelling can be a useful tool for exploring uncertainties about the natural history parameters of an infection, the temporal relationship between the infection and its sequelae, and the implications for sample size calculations in RCTs.

## Additional file

Additional file 1:
**Appendix for method and result section.**

## Abbreviations

Chlamydia: *Chlamydia trachomatis*
CI: Confidence interval
CrI: Credibility interval
PID: Pelvic inflammatory disease
POPI: Prevention Of Pelvic Infection trial
RCT: Randomised controlled trial
RR: Relative risk
