# Supplementary material for: Sample size considerations using mathematical models: an example with Chlamydia trachomatis infection and its sequelae pelvic inflammatory disease
Source: BMC Infect Dis. 2015 Jun 19;15:233. doi: 10.1186/s12879-015-0953-5 (PMC4472252; doi:10.1186/s12879-015-0953-5)
Supplement: Additional file 1: — Appendix for method and result section. [file 12879_2015_953_MOESM1_ESM.pdf]

Sample size considerations using mathematical models: an example with *Chlamydia trachomatis* infection and its sequelae pelvic inflammatory disease

Sereina A Herzog, Nicola Low, Andrea Berghold

Additional File 1

Table of Contents

List of Figures . . . 1
List of Tables . . . 1
1 Cumulative incidence of pelvic inflammatory disease (PID) . . . 2
2 Relative risk for the three types of progression to PID . . . 3
3 Sample size and relative risk . . . 4
4 The relation between sample size, relative risk and PID incidence while varying fraction developing PID . . . 5
4.1 Immediate progression and progression at the end . . . 5
4.2 Constant progression . . . 5
5 Sample size calculations used in the POPI trial . . . 6
5.1 Results of sensitivity analysis for scenario 1 . . . 6
6 Generic sample size calculation using the mathematical model . . . 10
6.1 Resulting RR and sample size for immediate progression varying duration of infection and fraction developing PID . . . 10
7 Including an immunity stage - a SIRS model . . . 11
7.1 Results . . . 11
8 References Additional File 1 . . . 15

List of Figures

S1 Figure S1: Influence of fraction f on sample size needed per group . . . 6
S2 Figure S2: Sensitivity analysis for scenario 1 - fraction f . . . 7
S3 Figure S3: Sensitivity analysis for scenario 1 - RR and sample size . . . 8
S4 Figure S4: Sensitivity analysis for scenario 1 - Difference in required sample sizes . . . 9
S5 Figure S5: Resulting RR and sample size for immediate progression varying duration of infection and fraction developing PID . . . 10
S6 Figure S6: Schematic overview of the model framework including an immunity stage . 11

List of Tables

S1 Table S1: Change prevalence or duration of infection and fix the other parameter . . . 7
S2 Table S2: Scenario 1 with SIRS model . . . 13
S3 Table S3: Scenario 2 with SIRS model . . . 14

1 Cumulative incidence of pelvic inflammatory disease (PID)

The function for the cumulative incidence of PID cases $C(t)$ at time point t depends for each type of progression (immediate progression, constant progression, progression at the end) on the specific incidence of PID cases and fulfills that $C(0) = 0$. The equations are similar to Herzog et al.[1]

$$\begin{aligned} C_{immediate}(t) &= f\lambda \int_0^t S(\tau) d\tau \\ &= \frac{f\lambda}{r+\lambda}\left((s+i_1+i_2)rt - \frac{(i_1+i_2)r - s\lambda}{r+\lambda} + \frac{e^{-(r+\lambda)t}((i_1+i_2)r - s\lambda)}{r+\lambda}\right) \end{aligned}$$

$$\begin{aligned} C_{constant}(t) &= \gamma \int_0^t I_1(\tau) d\tau \\ &= \frac{\gamma}{(r+\gamma)(\gamma-\lambda)(r+\lambda)}\Bigg((s+i_1+i_2)(\gamma-\lambda)\lambda rt \\ &+ \frac{\left(1-e^{-(r+\lambda)t}\right)(r+\gamma)\lambda(-(i_1+i_2)r + s\lambda)}{r+\lambda} \\ &+ \frac{\left(1-e^{-(r+\gamma)t}\right)(r+\lambda)\{i_1\gamma(r+\gamma) + (i_2 r - (s+i_1)\gamma)\lambda\}}{r+\gamma}\Bigg) \end{aligned}$$

$$\begin{aligned} C_{end}(t) &= fr \int_0^t \{I_1(\tau) + I_2(\tau)\} d\tau \\ &= \frac{fr}{r+\lambda}\left((s+i_1+i_2)\lambda t - \frac{-(i_1+i_2)r + s\lambda}{r+\lambda} + \frac{e^{-(r+\lambda)t}(-(i_1+i_2)r + s\lambda)}{r+\lambda}\right) \end{aligned}$$

where $s = S(0), i_1 = I_1(0)$ and $i_2 = I_2(0)$ describe the initial conditions for the constant progression scenario and satisfy the equation $s + i_1 + i_2 = 1$. The intervention group was treated for chlamydia infections and therefore has $s = 1, i_1 = 0$, and $i_2 = 0$. The control group starts at steady state in the absence of the intervention with $s = 1 - p, i_1 = p\frac{r}{r+\gamma} = p(1-f)$, and $i_2 = p\frac{\gamma}{r+\gamma} = pf$. The initial conditions for immediate progression and progression at the end of infection (where $\gamma = 0$ and $I(t) = I_1(t) + I_2(t)$) are described as $S(0) = s$ and $I(0) = i_1 + i_2$.

Remark: The cumulative PID incidence in the control group at time t equals for all three types of progression $frpt$. This is a consequence of our assumption that independent of the type of progression a certain fraction f of all infected women will develop PID in the absence of an intervention. We had to set the progression rate $\gamma = \frac{fr}{1-f}$ to achieve the same cumulative PID incidence in all three types of progression. The duration of infection in the constant progression type is $\frac{1}{r}$:

$$\begin{aligned} \text{Total mean duration being infected} &= \text{Mean duration in } I_1 + \\ &\quad \text{Probability going from from } I_1 \text{ to } I_2 \text{ times mean duration in } I_2 \\ &= \frac{1}{r+\gamma} + P[I_1 \text{to } I_2]\frac{1}{r} \\ &= \frac{1}{r+\gamma} + \frac{\gamma}{r+\gamma}\frac{1}{r} = \frac{r+\gamma}{(r+\gamma)r} = \frac{1}{r} \end{aligned}$$

2 Relative risk for the three types of progression to PID

The relative risk (RR) at time point $t > 0$ differs for each type of progression (immediate progression, constant progression, progression at the end) being dependent on the specific cumulative incidence of PID cases for the intervention group.

$$RR(t) = \frac{\text{cumulative PID incidence in the intervention group at time } t}{\text{cumulative PID incidence in the control group at time } t}$$

The cumulative PID incidence in the control group is the same for all three types of progression ($= frpt$), see section 1 on page 2. For the immediate progression and for the progression at the end, the RR is independent of the fraction f of infected women who develop PID:

$$RR_{immediate}(t) = \frac{f\lambda \int_0^t S(\tau)d\tau}{frpt} = \frac{p\left(1 - e^{-\frac{rt}{1-p}}\right) + rt}{rt}$$

$$RR_{end}(t) = \frac{fr \int_0^t \{I_1(\tau) + I_2(\tau)\}d\tau}{frpt} = \frac{e^{-\frac{rt}{1-p}}(1-p) - 1 + p + rt}{rt}$$

The RR depends on the fraction f for the constant progression because we set $\gamma = \frac{fr}{1-f}$ to achieve the same PID incidence in all processes in absence of the intervention (i.e. in the control group).

$$\begin{aligned} RR_{constant}(t) &= \frac{\gamma \int_0^t I_1(\tau)d\tau}{frpt} \\ &= \frac{1}{(f-p)rt}\left\{\left(1 - e^{-\frac{rt}{1-f}}\right)f^2 + \left(1 - e^{-\frac{rt}{1-p}}(1-p) - p - rt\right)p - \left(1 - e^{-\frac{rt}{1-f}} - rt\right)f\right\} \end{aligned}$$

3 Sample size and relative risk

Sample size needed per group is calculated using the method for the comparison of two proportions.[2]

$$n = \frac{\left\{u\sqrt{\pi_1(1-\pi_1)+\pi_0(1-\pi_0)} + v\sqrt{2\overline{\pi}(1-\overline{\pi})}\right\}^2}{(\pi_0-\pi_1)^2}$$

π_0	Proportion in control group who has the disease
π_1	Proportion in intervention group who has the disease
$\overline{\pi}$	$\frac{\pi_0+\pi_1}{2}$
u	One-sided percentage point of the normal distribution corresponding to 100% - power (e.g. power=90% $\longrightarrow u = 1.28$)
v	Percentage point of normal distribution corresponding to the (two-sided) significance level (e.g. if $\alpha = 0.05 \longrightarrow v = 1.96$)
n	Sample size per group

In our calculations, π_0 equals the cumulative incidence of PID cases in the control group and π_1 equals the cumulative incidence of PID cases in the intervention group at time t (i.e. at follow-up time). Using the relative risk (RR) with $RR = \frac{\pi_1}{\pi_0}$ yields

$$n = \frac{\left\{2u\sqrt{\pi_0[1+RR-\pi_0(1+RR^2)]} + v\sqrt{2\left[\pi_0(1+RR)(2-\pi_0-\pi_0 RR)\right]}\right\}}{4\pi_0^2(1-RR)^2}.$$

4 The relation between sample size, relative risk and PID incidence while varying fraction developing PID

We investigated what happens to the sample size needed per group if fraction f of women who develop PID is increased or decreased, respectively.

The sample size needed per group depends on the PID incidence in the control group and the relative risk (RR) - see section 3:

- The sample size needed per group is decreasing with increasing PID incidence in the control group and vice versa.
- The closer the RR is to 1 (i.e. no effect of intervention), the bigger is the sample size needed per group.

Note, the RR differs for the three types of progression (see section 2).

4.1 Immediate progression and progression at the end

For the immediate progression and for the progression at the end, the RR is independent of the fraction f of women who develop PID (see section 2). This means that changing fraction f influences the sample size needed per group only through the relationship between PID incidence and fraction f. The PID incidence in the control group is $= frpt$.

For $f \longrightarrow 0$: The sample size needed per group is increasing with decreasing f because PID incidence in the control group is decreasing and (if everything else is kept constant).

For $f \longrightarrow 1$: The sample size needed per group is decreasing with increasing f because PID incidence in the control group is increasing (if everything else is kept constant).

4.2 Constant progression

The relationship between sample size needed per group and fraction f is more complex for the constant progression. The PID incidence as well as the RR of the constant progression ($RR_{constant}$) depends on the fraction f (see section 2).

With the properties of $RR_{constant}$

- $RR_{constant}$ increases with increasing f;
- $RR_{constant} \xrightarrow{f \longrightarrow 1} RR_{immediate}$;
- $RR_{constant} \xrightarrow{f \longrightarrow 0} RR_{end}$;

we can conclude that there exists a f^* such that $RR_{constant} = 1$ because $RR_{immediate} \geq 1$ and $RR_{end} \leq RR_{immediate}$. Note, no analytical solution for f^* can be derived.

The PID incidence in the control group and the $RR_{constant}$ act in opposing directions if we look at the sample size needed per group for $f \longrightarrow f^*, f < f^*$ or $f \longrightarrow 0, f < f^*$:

- For $f \longrightarrow 0, f < f^*$: The $RR_{constant}$ decreases with $f \longrightarrow 0$ and the effect size $|1 - RR_{constant}|$ increases, i.e. sample size needed would decrease but the PID incidence decreases with $f \longrightarrow 0$ and hence sample size needed would increase.

- For $f \longrightarrow f^*, f < f^*$: The $RR_{constant}$ increases with $f \longrightarrow f^*$ and the effect size $|1 - RR_{constant}|$ decreases, i.e. sample size needed would increase but the PID incidence increases with $f \longrightarrow f^*$, i.e. sample size needed would decrease.

This considerations illustrate that there has to be a $f^{\#}$ at which the sample size needed is minimised. There are five distinction of cases (see **Figure S1**):

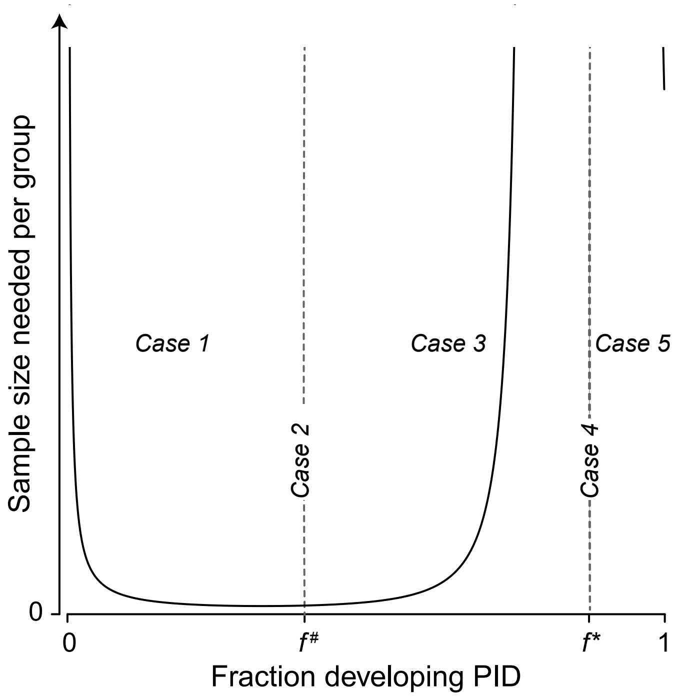

Figure S1: **Influence of fraction f on sample size needed per group.** There are five distinction of cases for the relationship between sample size needed per group and fraction f in the constant progression. The sample size is minimised at $f^{\#}$ and the $RR_{constant} = 1$ at f^*.

- **Case 1:** For $0 \leq f < f^{\#}$, the influence of the PID incidence is stronger than the $RR_{constant}$, i.e. sample size needed per group decreases for $f \longrightarrow f^{\#}$.
- **Case 2:** For $f = f^{\#}$, the sample size needed is minimised.
- **Case 3:** For $f^{\#} < f < f^*$, the influence of the PID incidence is weaker than the $RR_{constant}$, i.e. sample size needed per group increases for $f \longrightarrow f^*$.
- **Case 4:** For $f = f^*$, $RR_{constant} = 1$ and no sample size needed per group can be calculated due to the division by 0. Note, in this situation we would expect no effect from the intervention.
- **Case 5:** For $f^* < f \leq 1$, the $RR_{constant}$ increases and is bigger than 1 for $f \longrightarrow 1$ and the effect size $|1 - RR_{constant}|$ increases, i.e. sample size needed decreases and also the PID incidence increases which results in a decrease in sample size needed per group for $f \longrightarrow 1$, i.e. sample size needed per group decreases.

5 Sample size calculations used in the POPI trial

5.1 Results of sensitivity analysis for scenario 1

In scenario 1 of the POPI trial, assuming a 2% incidence of PID, a sample size of 2,115 women per group would allow the investigators to detect a RR of 0.48 (80% power, 5% significance level) using

the method for the comparison of two proportions (see section 3 on page 4).[3,4]

In the sensitivity analysis for scenario 1, the fraction f of women who develop PID in order to achieve the 2% PID incidence ranged between 15.9-80.3% at different levels of chlamydia prevalence and duration of infection, see **Figure S2**. The probability of multiple infections within the follow-up time decreases with increasing duration of infection, hence the fraction f has to increase in order to achieve the 2% PID incidence in the control group.

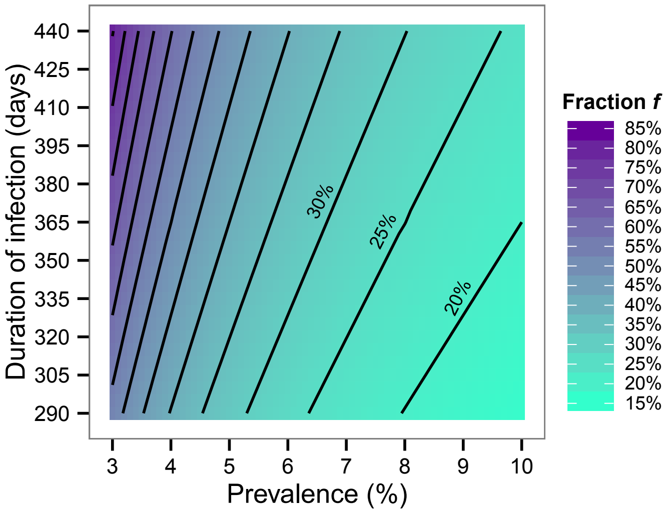

Figure S2: **Sensitivity analysis for scenario 1 - fraction *f*.** The fraction f of infected women who develop PID needed in order to achieve the 2% PID incidence in the control group.

For constant progression and progression at the end of the infection, changing the duration of infection influences the RR and the required sample size more than changing prevalence , see **Table S1** and **Figure S3**. The hypothesis of immediate progression was not investigated because the estimated RR was >1 in the main analysis .

For constant progression and progression at the end, **Table S1** shows the range of the RR and the sample size needed per group while prevalence is kept constant and duration of infection is varied or vice versa.

		**Fix p=7%, fix 1/r=365 days**	**Fix p=7%, vary 1/r in 290-440 days**	**Vary p in 3-10%, fix 1/r=365 days**
**Constant progression**	*RR*	0.49 (= 100%)	0.43 – 0.56 (88.5 – 115.2%)	0.47 – 0.50 (96.7 – 102.3%)
	N	2174 (= 100%)	1711 – 3036 (78.7 – 139.7%)	2040 – 2288 (93.8 – 105.3%)
**Progression at the end**	*RR*	0.39 (= 100%)	0.34 – 0.45 (87.8 – 116.2%)	0.38 – 0.40 (98.1 – 103.3%)
	N	1429 (= 100%)	1183 – 1869 (82.8 – 130.8%)	1366 – 1481 (95.6 – 103.6%)

Table S1: **Change prevalence** (p) **or duration of infection** $1/r$ **and fix the other parameter.** The first column shows the relative risk (RR) and the sample size needed per group (N) using the baseline values. The estimated RR and sample size needed per group values are then used as a 100% reference in the second and in the third column.

Figure S3 shows the RR and the required sample size for different levels of chlamydia prevalence and duration of infection. For constant progression, the estimated RR ranged between $0.42 - 0.57$ (**Figure S3**, Panel A) and the corresponding sample size needed per group between $1,618 - 3,223$

(**Figure S3**, Panel C). This range of sample size needed per group equals 76.5 – 152.4% of the POPI trial sample size calculation with 2% PID incidence and $RR = 0.48$ needing 2,115 women per group. For progression at the end, the estimated RR ranged between 0.33 – 0.46 (**Figure S3**, Panel B) and the corresponding sample size needed per group between $1,137 - 1,948$ (**Figure S3**, Panel D). This range of sample size needed per group equals $53.8 - 92.1\%$ of the POPI trial sample size calculation.

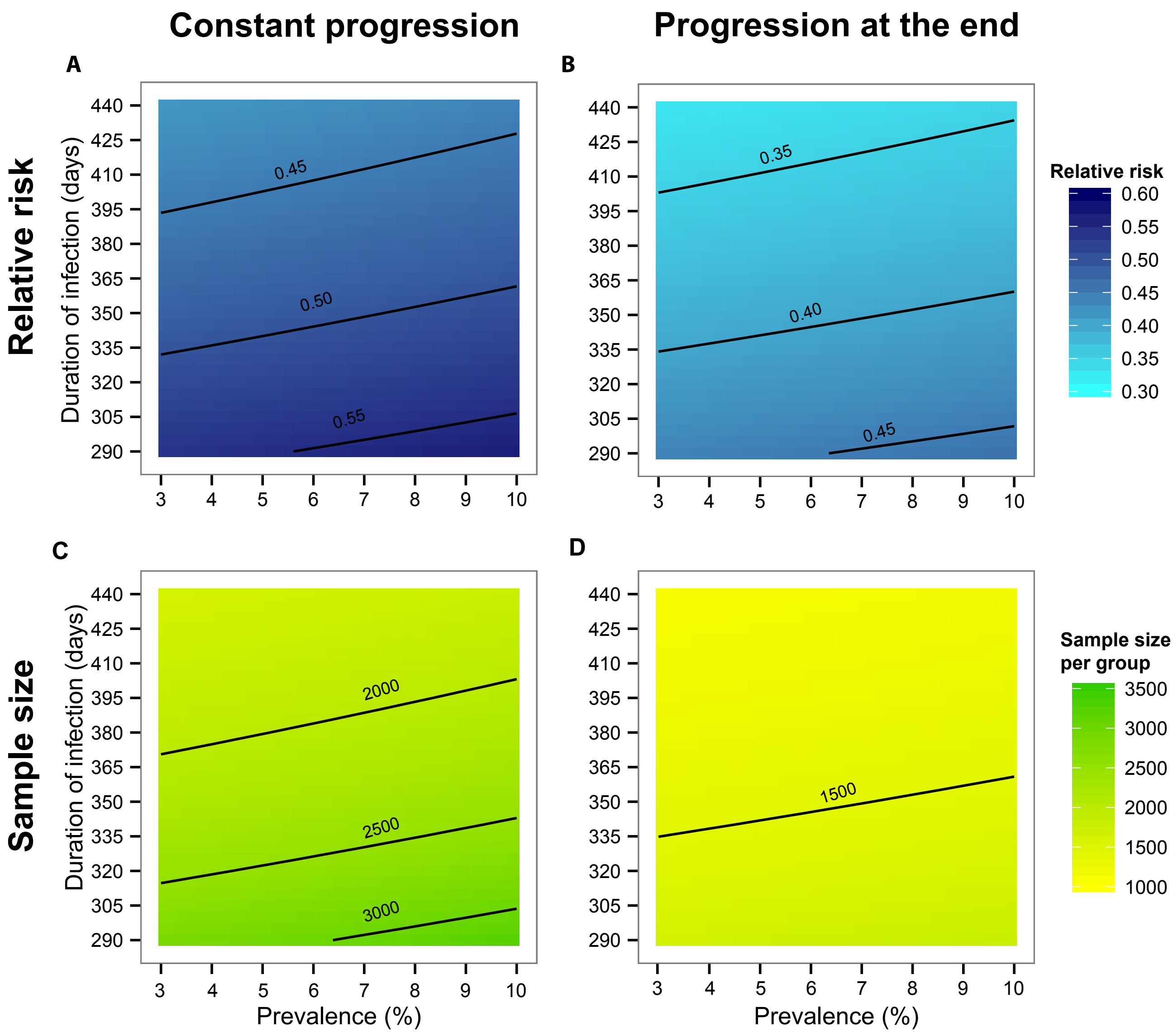

Figure S3: **Sensitivity analysis for scenario 1 - RR and sample size.** The RR and the corresponding sample size needed per group for constant progression (Panel A and C) and for progression at the end of infection (Panel B and D).

The sample size calculated with constant progression is closer to the POPI trial sample size calculation (2115 women per group) than with progression at the end of infection in 78.5% of the investigated combinations of prevalence and duration of infection. **Figure S4** shows the difference between the sample size calculated by constant progression and progression at the end relative to the POPI trial sample size calculation which uses 2% PID incidence with a RR=0.48. The difference ranged between -34.1% to 44.5%. A negative difference means that the sample size calculation from the constant progression is closer to the POPI trial sample size calculation than the sample size calculated by progression at the end. The sample size calculated with constant progression is closer to the POPI trial sample size calculation (2115 women per group) than with progression at the end of infection in 78.5% of the investigated combinations of prevalence and duration of infection. **Figure S4** shows the difference between the sample size calculated by constant progression and progression at the end relative to the POPI trial sample size calculation which uses 2% PID incidence with a RR=0.48. The difference ranged between -34.1% to 44.5%. A negative difference means that the sample size calculation from the constant progression is closer to the POPI trial sample size calculation than the sample size calculated by progression at the end.

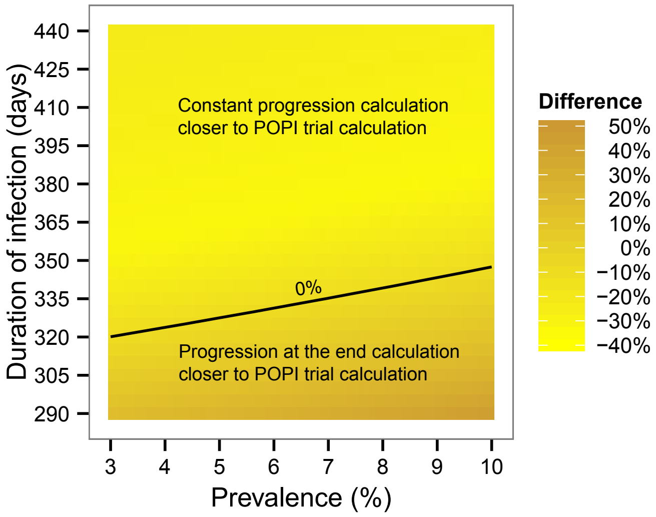

Figure S4: **Sensitivity analysis for scenario 1 - Difference in required sample size:** Difference in required sample sizes between constant progression and progression at the end relative to POPI trial calculation.

6 Generic sample size calculation using the mathematical model

6.1 Resulting RR and sample size for immediate progression varying duration of infection and fraction developing PID

The median RR is 1.046 (range 1.041-1.050) for the immediate progression model (**Figure S5**, Panel A) and the corresponding sample size needed per group has a median of 1,065,000 (range 812,000-1,589,000; **Figure S5**, Panel B).

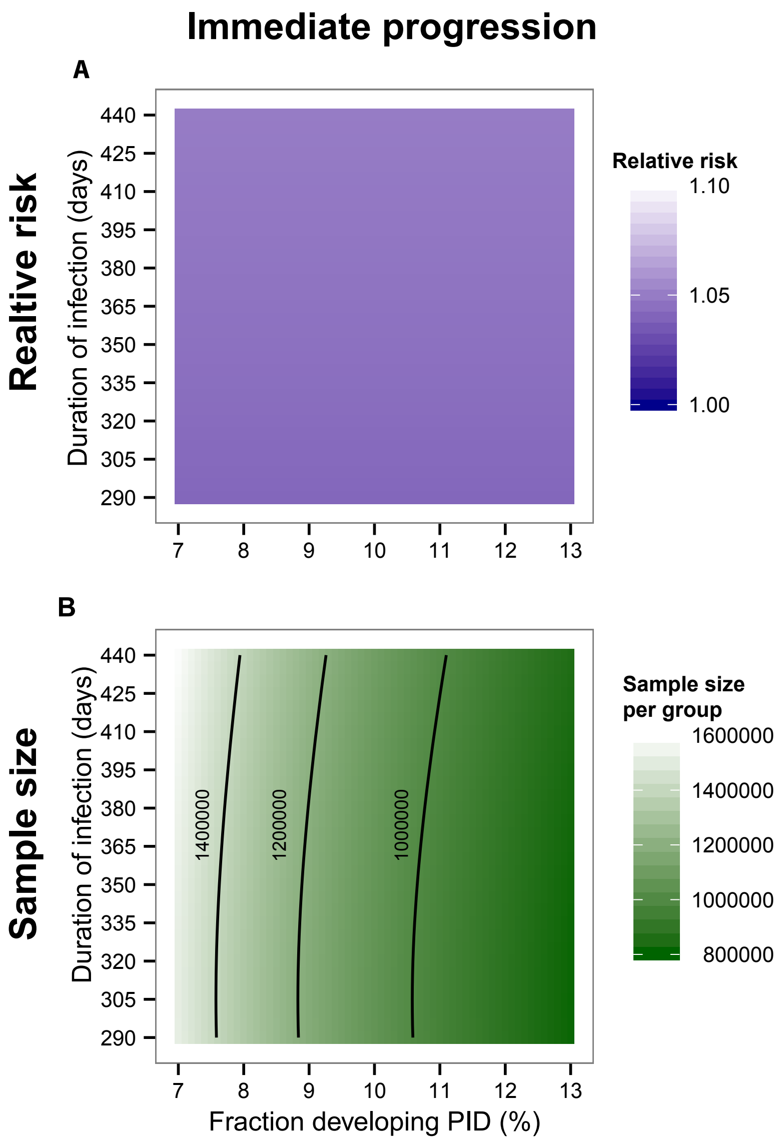

Figure S5: **Resulting RR and sample size for immediate progression varying duration of infection and fraction developing PID.**

7 Including an immunity stage - a SIRS model

There is an ongoing discussion about the existence and duration of immunity after a chlamydia infection.[5] We therefore investigated how our results for the sample size calculations used in the POPI trial would alter if we include an immunity stage. We used a Susceptible-Infected-Recovered-Susceptible (SIRS) compartmental model with which we investigated the same three hypothetical temporal relationship assumptions but having an immunity stage R included (see **Figure S6**). We denote with $1/\delta$ the duration of immunity.

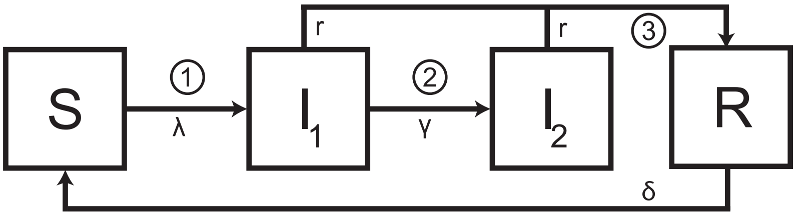

Figure S6: **Schematic overview of the model framework including an immunity stage.** The Susceptible-Infected-Recovered-Susceptible (SIRS) compartmental model allows investigating the three hypothetical temporal relationship assumptions having an immunity stage included with $1/\delta$ denoting the duration of immunity. Numbers indicate when during the chlamydia infection progression to PID could happen: 1) immediate progression, 2) constant progression, and 3) progression at the end.

We assumed that there is no immunity after treatment and that women in the immunity stage cannot be identified (i.e. not be differentiated from susceptible women or infected women). We did not consider that PID develops after chlamydia infection period. i.e. we investigated the same three types of progression: immediate progression, constant progression, and progression at the end.

The control group starts at steady state in the absence of the intervention with $S(0) = \frac{\delta - p(r+\delta)}{\delta}$, $I_1(0) = p\frac{r}{r+\gamma}$, $I_2(0) = p\frac{\gamma}{r+\gamma}$, and $R(0) = \frac{pr}{\delta}$.

The intervention group was treated for chlamydia infections but women in the immunity stage are not affected, i.e. they stay in the immunity stage: $S(0) = 1 - \frac{pr}{\delta}$, $I_1(0) = 0$, $I_2(0) = 0$, and $R(0) = \frac{pr}{\delta}$.

In order to observe a chlamydia prevalence p in absence of the trial we have to set $\lambda = -\frac{pr\delta}{pr-\delta-p\delta}$. We have to restrict the duration of immunity $1/\delta$ to allow only a positive constant force of infection λ:

$$\lambda > 0 \Leftrightarrow \delta > \frac{pr}{1-p} \Leftrightarrow \frac{1}{\delta} < \frac{1-p}{pr}$$

The duration of immunity has to be $< 4,849$ days $= 13.3$ years using the baseline values in Table 1 'Re-examine POPI trial' of the main paper. We investigated immunity duration between 0.25 and 1.5 years.

7.1 Results

The relative risk (RR) is increasing for all three types of progression with increasing duration of immunity (see **Table S2** and **S3** on p13-14). This means for constant progression and progression at the end that the effect size is decreasing and therefore sample size needed per group is increasing. For immediate progression, an increasing RR results in an increase in the effect size because RR> 1 and

therefore a decrease in the sample size needed per group. Note, a RR> 1 means that the predicted PID incidence in the intervention group is higher than in the control group.

In summary, including an immunity stage did not alter the results in our study about the sample size calculations used in the POPI trial:

- Scenario 1, assuming that PID can develop throughout the infection period (constant progression) results in relative risk (RR) values between 0.491 and 0.498 including an immunity stage. This is compatible with the RR=0.48 assumed by the POPI trial investigators for their first sample size calculation.
- Scenario 2, assuming that PID develops at the end of infection results in RR values between 0.391 and 0.397 including an immunity stage. This is closest to the RR=0.44 assumed by the POPI trial investigators for their second sample size calculation.

Duration of immunity (years)	Immediate progression			Constant progression			Progression at the end		
	PID incidence[a]	RR	Sample size per group	PID incidence[a]	RR	Sample size per group	PID incidence[a]	RR	Sample size per group
0	2.10	1.050	315,206	0.98	0.49	2,214	0.78	0.390	1,445
0.25	2.11	1.056	249,308	0.98	0.491	2,221	0.78	0.391	1,451
0.5	2.13	1.063	202,018	0.99	0.493	2,246	0.79	0.393	1,462
0.75	2.13	1.067	177,674	0.99	0.495	2,262	0.79	0.394	1,469
1	2.14	1.070	161,910	0.99	0.496	2,276	0.79	0.395	1,475
1.25	2.15	1.073	150,178	0.99	0.497	2,287	0.79	0.396	1,480
1.5	2.15	1.075	140,665	1.00	0.498	2,298	0.79	0.397	1,485

[a] in the intervention group (per year) in %.
PID, pelvic inflammatory disease; RR, relative risk.

Table S2: **Scenario 1 with SIRS model.** Analysing scenario 1 with 2% PID incidence in the control group and a relative risk (RR) of 0.48 using the SIRS model (80% power, 5% significance level). For the three types of progression we derived the PID incidence in the intervention group, the corresponding RR, and the sample size needed per group. Note, the first row with 0 years duration of immunity presents the results observed by the SIS model.

Duration of immunity (years)	Immediate progression			Constant progression			Progression at the end		
	PID incidence[a]	RR	Sample size per group	PID incidence[a]	RR	Sample size per group	PID incidence[a]	RR	Sample size per group
0	3.15	1.050	207,938	1.68	0.560	2,058	1.17	0.39	956
0.25	3.17	1.056	164,461	1.68	0.561	2,065	1.17	0.391	960
0.5	3.19	1.063	133,260	1.69	0.563	2,095	1.18	0.393	967
0.75	3.20	1.067	117,199	1.70	0.565	2,116	1.18	0.394	972
1	3.21	1.070	106,799	1.70	0.567	2,132	1.19	0.395	976
1.25	3.22	1.073	99,059	1.70	0.568	2,146	1.19	0.396	979
1.5	3.23	1.075	92,782	1.71	0.569	2,159	1.19	0.397	983

[a] in the intervention group (per year) in %.
PID, pelvic inflammatory disease; RR, relative risk.

Table S3: **Scenario 2 with SIRS model.** Analysing scenario 2 with 3% PID incidence in the control group and a relative risk (RR) of 0.44 using the SIRS model (80% power, 5% significance level). For the three types of progression we derived the PID incidence in the intervention group, the corresponding RR, and the sample size needed per group. Note, the first row with 0 years duration of immunity presents the results observed by the SIS model.

8 References Additional File 1

[1] Herzog, S.A., Althaus, C.L., Heijne, J.C.M., Oakeshott, P., Kerry, S., Hay, P., Low, N.: Timing of progression from Chlamydia trachomatis infection to pelvic inflammatory disease: a mathematical model. BMC Infectious Diseases **12**(1), 187 (2012)

[2] Kirkwood, B.R., Sterne, J.A.C.: Essential Medical Statistics, 2nd edn. Blackwell Science, Malden and Mass (2003)

[3] Oakeshott, P., Kerry, S., Aghaizu, A., Atherton, H., Hay, S., Taylor-Robinson, D., Simms, I., Hay, P.: Randomised controlled trial of screening for Chlamydia trachomatis to prevent pelvic inflammatory disease: the POPI (prevention of pelvic infection) trial. BMJ **340**, 1642 (2010)

[4] Oakeshott, P., Kerry, S., Atherton, H., Aghaizu, A., Hay, S., Taylor-Robinson, D., Simms, I., Hay, P.: Community-based trial of screening for Chlamydia trachomatis to prevent pelvic inflammatory disease: the POPI (prevention of pelvic infection) trial. Trials **9**, 73 (2008)

[5] Gottlieb, S.L., Martin, D.H., Xu, F., Byrne, G.I., Brunham, R.C.: Summary: the natural history and immunobiology of Chlamydia trachomatis genital infection and implications for chlamydia control. The Journal of infectious diseases **201**(Suppl 2), 190–204 (2010)
